# Validation of a Pseudovirus Neutralization Assay for Severe Acute Respiratory Syndrome Coronavirus 2: A High-Throughput Method for the Evaluation of Vaccine Immunogenicity

**DOI:** 10.3390/microorganisms12061201

**Published:** 2024-06-14

**Authors:** Zhaohui Cai, Raj Kalkeri, Mi Wang, Benjamin Haner, Dominic Dent, Bahar Osman, Paul Skonieczny, Jeremy Ross, Sheau-Line Feng, Rongman Cai, Mingzhu Zhu, Shane Cloney-Clark, Joyce S. Plested

**Affiliations:** Novavax, Inc., Gaithersburg, MD 20878, USA; zcai@novavax.com (Z.C.); rkalkeri@novavax.com (R.K.); mwang@novavax.com (M.W.); bhaner@novavax.com (B.H.); ddent@novavax.com (D.D.); bosman@novavax.com (B.O.); pskonieczny@novavax.com (P.S.); jross@novavax.com (J.R.); sfeng@novavax.com (S.-L.F.); rcai@novavax.com (R.C.); wcloneyclark@novavax.com (S.C.-C.)

**Keywords:** neutralization, pseudovirus, COVID-19, Omicron variant, emerging variants, XBB variants, validation, XBB.1.5, XBB.1.16, correlates of protection

## Abstract

The evaluation of coronavirus disease 2019 (COVID-19) vaccine immunogenicity remains essential as the severe acute respiratory syncytial virus 2 (SARS-CoV-2) pandemic continues to evolve and as additional variants emerge. Neutralizing antibodies are a known correlate of protection for SARS-CoV-2 vaccines. A pseudovirus neutralization (PNT) assay was developed and validated at Novavax Clinical Immunology Laboratories to allow for the detection of neutralizing antibodies in vaccine clinical trial sera. The PNT assay was precise, accurate, linear, and specific in measuring SARS-CoV-2 neutralization titers in human serum for ancestral strain and the Omicron subvariants BA.5 and XBB.1.5, with an overall geometric coefficient of variation of ≤43.4%, a percent relative bias within the expected range of −60% to 150%, and a linearity value of *R*^2^ > 0.98 for all three strains. This pseudovirus assay will be useful for the analysis of vaccine clinical trial samples to assess vaccine immunogenicity. Future work will focus on modifying the assay for emerging variants, including XBB.1.16, EG.5.1, BA.2.86, and any other variants that emerge in the ongoing pandemic.

## 1. Introduction

The coronavirus disease 2019 (COVID-19) pandemic was caused by the severe acute respiratory syncytial virus 2 (SARS-CoV-2) virus [1]. New variants continue to emerge and circulate, such as Omicron XBB.1.16, EG.5.1, HV.1, etc., leading to the sustained transmission of the virus and clinical infections. Earlier ancestral/prototype COVID-19 vaccines may not be as effective against variant SARS-CoV-2 because some variants (such as the Omicron subvariants) have developed immune evasion properties [2,3,4]. There is a continued need for high-throughput, robust, cost-effective, and flexible methods that can be used to assess the immunogenicity of vaccines [5], especially with emerging variants and the continued use of updated vaccine boosters.

Antibodies against SARS-CoV-2, particularly against spike (S) protein, protect against infection [6]. Anti-S neutralizing antibodies inhibit infection by binding to viral S protein and interrupting binding to the cell receptor human angiotensin-converting enzyme 2 (hACE2) [6,7,8]. Neutralizing antibodies are a known correlate of protection (CoP) for vaccines [8].

Pseudoviruses are engineered to contain genetic components of the original SARS-CoV-2 virus, but they lack the parts needed for sustained replication [9]. This article describes the validation of an in vitro pseudovirus-based neutralization (PNT) assay to detect and quantitate neutralizing antibodies against SARS-CoV-2 ancestral and variant strains (Omicron BA.5 and Omicron XBB.1.5) at Novavax Clinical Immunology Laboratories. The assay was assessed for precision, specificity, linearity, and other validation parameters. The geometric mean titer (GMT) for the ancestral strain was normalized to the World Health Organization (WHO) international standard in international units (IU/mL) for the calibration and harmonization of the neutralization assay.

The PNT assay is a robust, high-throughput, and rapid method used for detecting neutralizing antibodies, which are a CoP for vaccine efficacy. This validated method is suitable for use in the testing of clinical trial sera.

## 2. Materials and Methods

### 2.1. Assay Procedure

**Figure 1:**
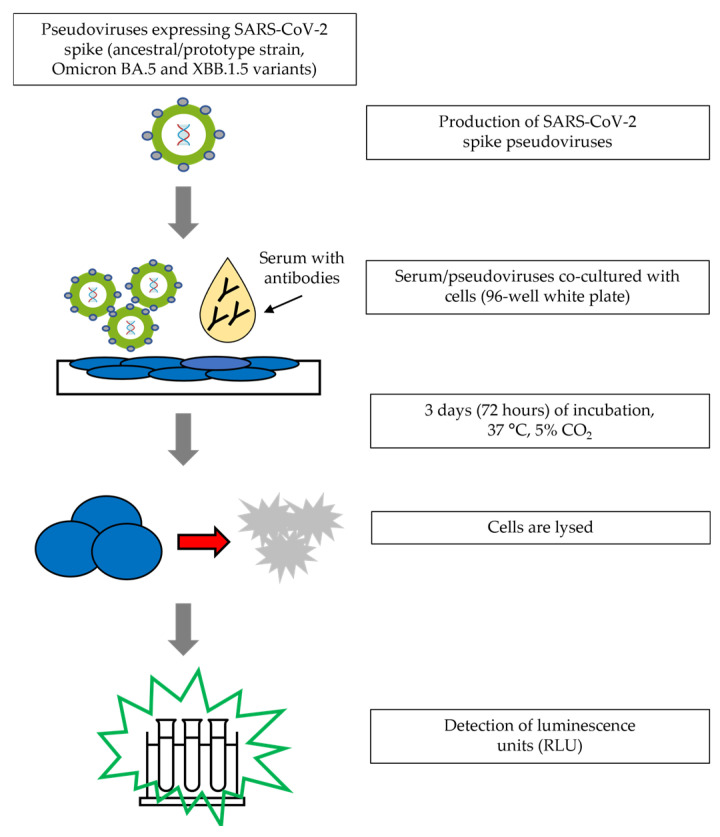
Pseudovirus assay testing procedure.

A PNT assay was developed to assess virus-specific neutralizing antibodies against SARS-CoV-2 in human serum [10]. To perform the assay, serum samples were heat-inactivated in a 56 °C water bath for 30 min prior to the assay. Serum samples were serially diluted in an infection medium in a 96-well white opaque cell culture plate. The working dilution of the pseudovirus (eEnzyme, LLC, Gaithersburg, MD, USA) was then added to each well, before being incubated at 37 °C for 2 h. Then, HEK293T/hACE2 cells (obtained from Creative Biogene, Shirley, NY, USA) were added to the wells, before being incubated for 72 h at 37 °C. After incubation, the Bright-Glo™ luciferase substrate (Promega, Madison, WI, USA) was added to each well. Plates were incubated for 15 min at room temperature without ambient light. Viral entry into the cells was determined by measuring the luminescence with a SpectraMax^®^ iD3 microplate reader (Molecular Devices, San Jose, CA, USA). Neutralization titers represent the inhibitory dilution of serum samples at which relative luminescence units (RLUs) were reduced by 50% compared to virus control wells on each plate. Data were analyzed and 50% pseudovirus neutralization titers (ID_50_) were calculated using 4-parameter curve fitting in SoftMax^®^ Pro software 7.1 (Molecular Devices, San Jose, CA, USA) (Figure 1). The assay was originally developed using the ancestral pseudovirus strain but was later modified for Omicron BA.5 and Omicron XBB.1.5 pseudovirus.

### 2.2. Samples

Up to 32 human serum samples tested in the PNT assay came from commercial sources (BioIVT, Westbury, NY, USA, or Biological Specialty Co., Reading, PA, USA), the Novavax phase 3 influenza vaccine trial (qNIV-E-301), COVID-19 vaccine trial (2019nCoV-311 part 2), the Novavax SARS-CoV-2 vaccine trial (2019nCoV-501), or the Novavax respiratory syncytial virus (RSV) phase 3 trial (RSV-M-301) (clinical samples from Novavax, Inc., Gaithersburg, MD, USA).

Positive quality control (QC) samples known to have high (HQC), medium (MQC), or low (LQC) PNT titers and negative controls (NCs) were either purchased from a commercial source (BioIVT) sera or prepared in house by mixing high-titer human serum with NC.

For the analysis of conversion to WHO international standard units, the WHO international standard (NIBSC code 20/136) was used in the ancestral strain PNT assay.

### 2.3. Validation Assays

#### 2.3.1. Precision

Precision was assessed using a panel of 40 serum samples that had a range of negative to high titers in the PNT assay. The PNT assay was performed in six runs by two analysts on three different days, with each sample tested twice per run. The percent geometric coefficient of variation (%GCV) was calculated based on variance component analysis using a sample as a fixed effect and the analyst and the day as random effects. The %GCV was then used to describe the precision (total, intra-assay, and inter-assay).

#### 2.3.2. Specificity

The PNT assay was performed on samples collected prior to the SARS-CoV-2 pandemic, which were expected to cover the lower limit of quantification (LLoQ). Additionally, five pairs of pre- and post-vaccination sera from phase 3 trials of influenza or RSV vaccines (Novavax, Inc.) were assessed in the PNT assay. These sample pairs were expected to have <LLoQ PNT results while showing strong specific immune responses to influenza or RSV post-vaccination, demonstrating the specificity of the PNT assay.

#### 2.3.3. Linearity

Two SARS-CoV-2 PNT-positive samples were tested in the PNT assay undiluted or at various dilutions in negative serum. The samples were tested twice in the same run in duplicates across six runs by two different analysts on three different days. Linear regression was then performed on the sample results and the percent relative bias was calculated as follows:

(1) $$\%\;Relative\;Bias=100\times \frac{Observed\;overall\;PNT\;GMT-Expected\;PNT\;Titer}{Expected\;PNT\;Titer}$$

#### 2.3.4. Sensitivity and Analytical Range

The lowest and highest PNT titers that could be quantified with acceptable precision and accuracy (as above) were defined as the LLoQ and upper limit of quantitation (ULoQ), respectively. The LLoQ provides information about assay sensitivity.

#### 2.3.5. Conversion to WHO International Standard Units (IU/mL)

To allow conversion between the assay units (PNT titers) and WHO international standard units (IU/mL), WHO international standard for the anti–SARS-CoV-2 immunoglobulin (human) ancestral strain (NIBSC code 20/136) was tested during validation. From the results, a conversion formula from PNT ID50 to IU/mL was defined.

### 2.4. Variant Assays

Although the PNT assay was first developed for the ancestral strain of SARS-CoV-2, the emergence of variants necessitated the adaptation of the assay for variant strains. The PNT assay was extended for the Omicron XBB.1.5 and Omicron BA.5 variants. A validation process similar to that of the ancestral strain was used for these variant assays.

### 2.5. Correlation Analyses

Serum samples were assessed in the PNT (with the same methodology used for validation) and live wild-type virus microneutralization (MN) assays [11]. Linear regression and Bland–Altman assay analysis were conducted using GraphPad Prism^®^ software (San Diego, CA, USA; Version 9.3.1) and SAS software (v9.4, SAS Institute Inc., Cary, NC, USA) to determine the correlation between the PNT and MN assay results.

## 3. Results

### 3.1. Ancestral Strain Validation Parameters

The acceptance criterion for precision was ≤60% GCV for the ancestral strain, and <50% for BA5 and XBB.1.5 for at least 80% of the tested samples. The overall ancestral strain inter-assay, intra-assay, and total assay %GCV values were 6.6%, 42.8%, and 43.4%, respectively (Table 1). A total of 95% (38 of 40), 90% (36 of 40), and 65% (26 of 40) of samples exhibited inter-assay, intra- assay, and total %GCV values less than 50%, respectively. A total of 97.5% (39 of 40), 100% (40 of 40), and 85% (34 of 40) of samples exhibited inter-assay, intra-assay, and overall %GCV values less than or equal to 60%, respectively.

The specificity of the PNT assay was successfully demonstrated (Table 2). Human serum collected prior to the SARS-CoV-2 pandemic era had no detectable titer for the Wuhan D614 virus (<LLoQ). The LLoQ was determined based on the lowest titer values that were accurately and precisely detected for the sample, as mentioned in the Methods section (based on the acceptance criteria established in the validation protocol). The LLoQ represents the lowest quantity of the analyte that can be accurately, precisely, and reproducibly quantitated in the assay.

**Figure 2:**
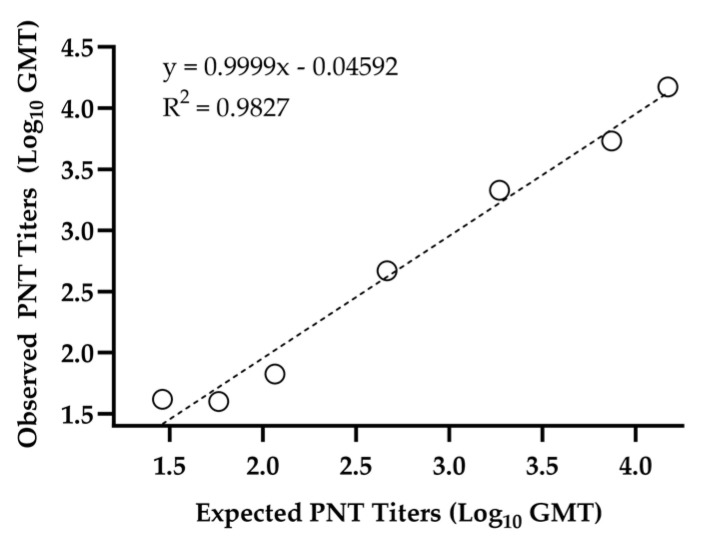
Pseudovirus assay ancestral strain linearity of a positive sample. The positive sample was diluted in series and tested in six assay runs. Linearity was then evaluated by calculating the precision and accuracy of the titer at each dilution, and the slope of linear regression lines for each sample. Sample with only values above the LLoQ are presented in the figure. GMT, geometric mean titer; PNT, pseudovirus neutralization.

The five pairs of pre- and post-vaccination sera from RSV- or influenza-vaccinated participants (pre-pandemic) all tested negative (<LLoQ) in the ancestral PNT assay. These samples had strong specific immune responses to influenza or RSV post-vaccination, demonstrating the specificity of the SARS-CoV-2 PNT assay. The linearity of the assay was successfully demonstrated, with an R^2^ value of 0.983 for the individual positive samples that were examined (Figure 2 and Table 3 and Table 4).

The linearity of the assay was successfully demonstrated, with an *R*^2^ value of 0.983 for the individual positive samples that were examined (Figure 2 and Table 3 and Table 4).

The LLoQ was assigned as a GMT of 42, based on the lowest titer values that were accurately and precisely detected for the sample (as well as the precision of the assay shown in Table 4). Based on the available tested samples, the ULoQ was found to be a titer of at least 14,863 for the ancestral strain.

The WHO international standard for anti–SARS-CoV-2 immunoglobulin (human) (NIBSC code: 20/136) was also tested during validation. The WHO-assigned concentration of 20/136 was 1000 IU/mL. The overall GMT of 20/136 from validation was 4680.9. Therefore, the conversion factor of 0.214 (1000/4680.9) was determined to be used as an internal reference for assay calibration and harmonization (PNT titer * 0.214 = IU/mL).

### 3.2. Assay Validations for Variants

**Figure 3:**
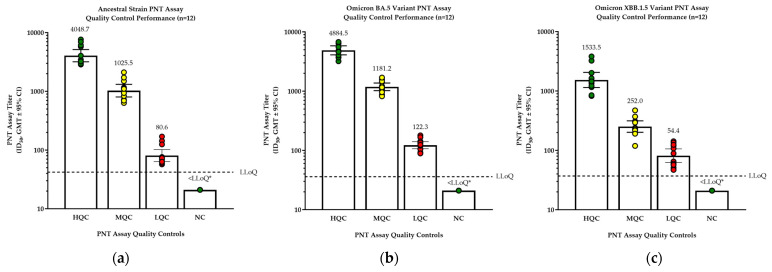
Pseudovirus assay ancestral strain and variants’ quality control standards performance. For (**a**) the ancestral strain, (**b**) the Omicron BA.5 variant, or (**c**) the Omicron XBB.1.5 variant, positive quality control (QC) samples were tested in the pseudovirus assay to examine assay performance. * Values for NC that were <LLoQ were plotted as 0.5x LLoQ. GMT, geometric mean titer; HQC, positive QC samples with high PNT titers; LLoQ, lower limit of quantification; LQC, positive QC samples with low PNT titers; MQC, positive QC samples with medium PNT titers; NC, negative control; PNT, pseudovirus neutralization.

The original assay was developed using the ancestral strain and later adapted to variant strains (including Omicron BA.5 and Omicron XBB.1.5). Quality control samples performed similarly for Omicron variants and the ancestral strain (Figure 3).

Results for the assay validation parameters for the Omicron variants were similar to those for the ancestral strain (Supplementary Table S1). For Omicron BA.5, inter-assay, intra-assay, and total precision exhibited a %GCV ≤ 50% in 95%, 100%, and 97.5% of the samples, respectively. All pre-COVID healthy human samples were <LLoQ; all RSV- or influenza-vaccinated sera were <LLoQ for SARS-CoV-2 while showing strong specific responses to RSV or influenza. Linearity was successfully demonstrated (*R*^2^ = 0.987 and 0.984), the LLoQ was defined as 36, and the ULoQ was determined to be at least 15,856.

For Omicron XBB.1.5, inter-assay, intra-assay, and total precision exhibited %GCV ≤50% in 80%, 97.5%, and 95% of samples, respectively. All pre-COVID healthy human samples were <LLoQ; all RSV- or influenza-vaccinated sera were <LLoQ for SARS-CoV-2 while showing strong specific responses to RSV or influenza. Linearity was successfully demonstrated (*R*^2^ = 0.981 and 0.989), the LLoQ was defined as 37, and the ULoQ was determined to be at least 7561.

### 3.3. Correlation between the PNT Assay and the Whole-Virus-Based MN Assay

**Figure 4:**
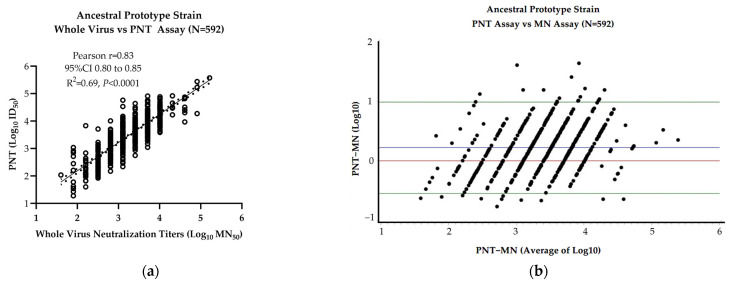
Neutralization titers from whole wild-type virus (MN_50_) and pseudovirus (ID50)-based neutralization assays from N = 592 serum samples (including both anti–NP-positive and -negative) were subjected to correlation analysis for comparison purposes using GraphPad Prism (v9.3.1) (**a**) and Bland–Altman analysis (**b**). Pearson *r* values with a 95% confidence interval, *R*^2^ values, and two-tailed *P* values are shown in the graph. The solid line shows the regression trend and dotted lines show a 95% CI of the regression trend (**a**). In Figure (**b**), the red horizontal line shows a perfect agreement between the PNT and MN assays, the blue horizontal line shows the average log 10 difference between the PNT and MN assays, and the green horizontal lines show the average log10 difference ± 2 standard deviations.

A systematic comparison between the whole wild-type-virus-based MN and PNT assays was performed. The PNT assay method used the same method as that used for validation (before validation). The data include all participants (both anti–NP-positive and -negative at baseline) at three time points (days 1, 14, and 28) with a total of N = 592 samples. A significant correlation of 50% neutralization titers between the two assays (N = 592, Pearson *r* = 0.83, *p* < 0.0001) was observed (Figure 4a). This is in line with the correlation reported in papers by Sholukh et al. [12] and Bhiman JN et al. [13] that reported a Pearson *r* = 0.81–0.89 using similar cell-based whole-virus assays to draw comparisons with the PNT assay. In order to understand the bias between the PNT and MN assays, the Bland–Altman assay was performed (Figure 4b). This analysis revealed a small bias of 0.225 (95% limits of agreement from –0.54 to 0.98) and the majority of the data fell within two standard deviations of the mean, further confirming the initial correlation analysis. Our correlation results, along with the literature data, confirm the similarity between both the whole-virus and pseudovirus neutralization assays and therefore indicate no significant difference in the neutralizing antibody results (Figure 4a,b) from either of the assays.

Our previous publication [10] was an FFP method paper, whereas this manuscript focuses on method validation. The principle of the assay mentioned in both manuscripts is the same. At the time of Cai Z et al.’s publication, the PNT assay was evolving, and we were still focused on developing/adapting the assay for other emerging variants in parallel (evaluating qualification data, etc.). Since Cai Z et al.’s publication, we have further fine-tuned the PNT methodology, leading to this validation effort. Some of the major differences between Cai Z et al.’s assay and the validated assay in this study include a longer assay duration (3 days in this study vs. 2 days in Cai Z et al.’s), a difference in the input virus (previously based on TCID50, but in this study, based on 50,000 RLU), and a difference in the backbone (lentivirus vs. the murine leukemia virus (MLV)).

## 4. Discussion

The PNT assay described herein showed accuracy, linearity, and specificity for SARS-CoV-2 neutralizing antibodies in human serum. The PNT assay was developed for the ancestral strain (Wuhan), as well as the Omicron subvariants BA.5 and XBB.1.5. As the IU/mL conversion factor was determined, results from the assay for the ancestral strain can also be converted to IU/mL, allowing comparisons to be drawn with other standardized assays and CoP levels, as well as comparisons with the data generated in other laboratories. These results suggest that the PNT assay will be useful for the measurement of neutralization in clinical trial samples for vaccine development.

The PNT assay provides a high-throughput, robust, and cost-effective method for the BSL-2 laboratory that can be used to assess neutralizing antibodies, which are a known correlate of protection [14]. The pseudovirus assay was not only precise and accurate for the ancestral strain, but also for Omicron subvariants (BA.5 and XBB.1.5). This is critical as some Omicron subvariants have shown immune evasion properties [15], and the assessment of vaccine immunogenicity against these strains is important for determining the potential effectiveness of the vaccine with the updated strains.

Close similarity between whole virus- and pseudovirus-based neutralization assays has been well documented in the literature [12,16,17]. Significant correlation (Pearson *r* = 0.83, *p* < 0.0001) and Bland–Altman analyses between the pseudovirus neutralization (conducted in a biosafety laboratory (BSL)-2) and whole-virus neutralization (conducted in BSL-3) assays confirm the concordance between the two assays. Our correlation data are in line with the correlation reported in Sholukh et al.’s [12] and Bhiman JN et al.’s [13] papers that reported Pearson *r* = 0.81–0.89 using similar cell-based whole-virus assays to draw comparisons to the PNT assay.

The pseudovirus system employed in this PNT assay is particularly useful for clinical studies, CoP studies, and the assessment of emerging variants [9]. This is, in part, because the PNT assay described is robust, high-throughput, and can be performed under less restrictive BSL conditions (BSL-2 compared to BSL-3). To our knowledge, this is the first report of a PNT assay for the XBB.1.5 variant. As the XBB.1.5 variant is currently circulating (at the time of writing), our validated assay will be useful for the evaluation of XBB.1.5 strain-based monovalent vaccine boosters, recently recommended by the FDA [18] and the WHO [19].

In contrast to the ELISA-based measurements of either anti-rS antibodies or hACE2 binding inhibition (SARS-CoV-2 receptor binding inhibition) assays, this is a cell-based assay, involving additional assay set-up and incubation time, leading to a longer data turnaround time. Although the CoP levels for emerging variants using PNT still need to be established, it may be possible to determine them, considering that the CoP levels for the ancestral strain have already been established [14]. As of now, the harmonization of pseudovirus assays for currently circulating XBB variants is difficult due to the current lack of suitable international standards for Omicron variants (XBB.1.5).

Compared to most of the laboratories, we used the MLV system for PNT in our assays. This assay system might be less affected by HIV antivirals (which might inhibit lentivirus based PNT assays and artificially show high neutralization titers) and might be more suitable for testing serum from subjects treated with HIV antivirals. We added this to the Discussion section. A limitation of this assay methodology is that the ULoQ was based on sufficiently high-titer serum samples available during the time of assay validation. Along similar lines, the adaptability of the validation method to forward-drifted variants is based on the availability of serum samples with reasonably high titers to establish the ULoQ. Other limitations of the PNT assay, already mentioned in our previous publication [10], include the need for special critical reagents (such as pseudoviruses expressing the S protein), the permissive cell lines, and the fact that the pseudovirus may not mimic the distribution/presentation of antigens presented on the live virus.

## 5. Conclusions

The PNT assay demonstrated accuracy, linearity, and specificity in measuring neutralization titers in human serum for the ancestral strain and the Omicron subvariants BA.5 and XBB.1.5. This pseudovirus assay will be a useful high-throughput and BSL-2–compatible method for analyzing vaccine clinical trial samples to assess vaccine immunogenicity. Future work will focus on adapting the assay for emerging variants, including XBB.1.16 and any other variants that emerge.

## 6. Patents

The data reported in this manuscript are disclosed in a patent application.

## Figures and Tables

**Table 1:** Pseudovirus assay ancestral strain precision.

Sample	N ^1^	PNT Titer GMT ^2^	Inter-Assay %GCV	Intra-Assay %GCV	Total %GCV
Overall ^3^	N/A	N/A	6.6	42.8	43.4
1	24	4048.7	46.6	34.2	60.0
2	24	1025.5	12.9	42.4	44.7
3	24	80.6	39.8	33.2	53.4
4	24	20.0^2^	0.0	0.0	0.0
5	24	104.0	0.0	35.9	35.9
6	24	281.8	10.9	25.5	27.9
7	24	12,248.5	9.0	35.5	36.8
8	24	172.3	0.0	41.8	41.8
9	24	193.9	0.0	40.4	40.4
10	24	11,589.0	6.6	40.7	41.4
11	24	8463.1	12.4	36.2	38.6
12	24	2128.9	25.9	56.4	63.7
13	23	28.0	0.0	49.4	49.4
14	24	816.0	7.3	37.8	38.6
15	24	2424.7	13.8	34.5	37.5
16	24	397.0	0.0	43.9	43.9
17	24	2269.5	10.6	43.6	45.1
18	24	375.9	13.5	38.4	41.0
19	24	783.7	3.6	43.2	43.4
20	24	1978.5	44.8	49.4	70.3
21	24	1322.5	18.1	42.9	47.2
22	24	3044.9	50.8	41.0	68.5
23	24	5885.3	38.9	36.3	55.1
24	24	1330.1	0.0	52.9	52.9
25	24	546.9	37.8	33.0	51.7
26	22	230.2	27.5	25.2	37.9
27	18	73.0	60.5	39.6	76.1
28	21	27.8	17.8	36.6	41.2
29	22	20.4	3.0	4.7	5.6
30	24	32.8	24.6	50.6	57.6
31	24	34.4	45.8	49.3	71.0
32	24	14,863.5	0.0	59.8	59.8
33	24	5386.6	47.2	42.6	66.7
34	24	2136.1	7.6	43.5	44.3
35	24	468.2	0.0	40.7	40.7
36	21	66.0	0.0	23.5	23.5
37	19	44.1	0.0	41.1	41.1
38	23	42.8	0.0	46.1	46.1
39	24	38.1	31.8	46.4	58.1
WHO Reference 20/136	16	4680.9	0.0	49.0	49.0

^1^ Number of values used for calculation. ^2^ When the PNT titer was <20 (MRD), a value of 20 was used for calculation purposes. ^3^ The overall assay precision with Wuhan D614 was calculated at the strain level by taking into account all 40 individual samples listed in the table. Note: GCV = geometric coefficient of variation; GMT = geometric mean titer.

**Table 2:** Pseudovirus assay ancestral strain assay specificity.

Pre-COVID-19 healthy human sera							
**Human** **serum**				**PNT titer**			
Positive Control-1				4048.7			
Positive Control-2				1025.5			
Positive Control-3				80.6			
Pre-COVID-1				<LLoQ			
Pre-COVID-2				<LLoQ			
Pre-COVID-3				<LLoQ			
Pre-COVID-4				<LLoQ			
Pre-COVID-5				<LLoQ			
Pre-COVID-6				<LLoQ			
Pre-COVID-7				<LLoQ			
Pre-COVID-8				<LLoQ			
**RSV F protein-vaccinated sera**							
**Participant** **ID**	**Anti-RSV F protein antibody**				**Wuhan D614 prototype PNT titer**		
	**Pre-RSV F vaccination** **(day 0)**	**Post-RSV F vaccination** **(day 14)**	**Post/pre ratio**		**Pre-RSV F vaccination** **(day 0)**	**Post-RSV F vaccination** **(day 14)**	**Post/pre ratio**
41	509	43,767	86		<LLoQ	<LLoQ	1
42	474	20,188	42.6		<LLoQ	<LLoQ	1
43	483	9721	20.1		<LLoQ	<LLoQ	1
44	632	10,958	17.3		<LLoQ	<LLoQ	1
45	772	15,869	20.6		<LLoQ	<LLoQ	1
**Influenza-vaccinated sera**							
**Participant ID**	**Influenza HAI titer (A/Kansas/14/2017) (H3N2)**				**Wuhan D614 prototype PNT titer**		
	**Pre-influenza vaccination** **(day 0)**	**Post-influenza vaccination** **(day 28)**	**Post/pre-vaccination ratio**		**Pre-influenza vaccination** **(day 0)**	**Post-influenza vaccination** **(day 28)**	**Post/pre-vaccination ratio**
46	10	640	64		<LLoQ	<LLoQ	1
47	20	1280	64		<LLoQ	<LLoQ	1
48	10	1280	128		<LLoQ	<LLoQ	1
49	20	2560	128		<LLoQ	<LLoQ	1
50	40	2560	64		<LLoQ	<LLoQ	1

COVID-19, coronavirus disease 2019; HAI, hemagglutination inhibition; LLoQ = lower limit of quantitation; PNT, pseudovirus neutralization; RSV, respiratory syncytial virus.

**Table 3:** Pseudovirus assay ancestral strain linearity.

Parameter	Estimate	95% LCL	95% UCL
Slope	1.000	0.847	1.153
Intercept	−0.046	−0.492	0.400
*R* ^2^	0.983	N/A	

LCL, lower confidence limit; UCL, upper confidence limit.

**Table 4:** Pseudovirus assay ancestral strain dilution results.

Dilution	N	Observed PNT GMT	Expected PNT GMT	%Relative Bias	Inter-Assay %GCV	Intra-Assay %GCV	Total %GCV
1	12	14,863.2	14,863.2	0.0	0.0	59.8	59.8
2	12	5386.6	7431.6	−27.5	47.2 ^1^	42.6	66.7
8	12	2136.2	1857.9	15.0	7.6	43.5	44.3
32	12	468.2	464.5	0.8	0.0	40.7	40.7
128	12	66.7	116.1	−42.6	0.0	23.5	23.5
256	12	39.9	58.1	−31.3	0.0	41.1	41.1
512	12	41.6	29.0	43.4	0.0	46.1	46.1
1024	12	38.0	14.5	161.9	31.8 ^1^	46.4	58.1

GCV, geometric coefficient of variation; GMT, geometric mean titer; PNT, pseudovirus neutralization. ^1^ Higher inter-assay %GCV for dilutions 2 and 1024 seems to be a random variation that might be biological in nature.

## Data Availability

The data presented in this study are available on request from the corresponding author. The data are not publicly available due to the proprietary subject and sample information.

## Supplementary Materials

The following supporting information can be downloaded at: https://www.mdpi.com/article/10.3390/microorganisms12061201/s1. Table S1: Pseudovirus assay results for SARS-CoV-2 variant strains.

## References

[1] Centers for Disease Control and Prevention David J. Spencer CDC Museum—COVID-19 Timeline. https://www.cdc.gov/museum/timeline/covid19.html.

[2] Fendler A., de Vries E.G.E., GeurtsvanKessel C.H., Haanen J.B., Wormann B., Turajlic S., von Lilienfeld-Toal M. (2022). COVID-19 vaccines in patients with cancer: Immunogenicity, efficacy and safety. Nat. Rev. Clin. Oncol..

[3] Fiolet T., Kherabi Y., MacDonald C.J., Ghosn J., Peiffer-Smadja N. (2022). Comparing COVID-19 vaccines for their characteristics, efficacy and effectiveness against SARS-CoV-2 and variants of concern: A narrative review. Clin. Microbiol. Infect..

[4] Liu Y., Rocklov J. (2022). The effective reproductive number of the Omicron variant of SARS-CoV-2 is several times relative to Delta. J. Travel. Med..

[5] Van Tilbeurgh M., Lemdani K., Beignon A.S., Chapon C., Tchitchek N., Cheraitia L., Marcos Lopez E., Pascal Q., Le Grand R., Maisonnasse P. (2021). Predictive Markers of Immunogenicity and Efficacy for Human Vaccines. Vaccines.

[6] Pang N.Y., Pang A.S., Chow V.T., Wang D.Y. (2021). Understanding neutralising antibodies against SARS-CoV-2 and their implications in clinical practice. Mil. Med. Res..

[7] Jackson C.B., Farzan M., Chen B., Choe H. (2022). Mechanisms of SARS-CoV-2 entry into cells. Nat. Rev. Mol. Cell Biol..

[8] Misra A., Theel E.S. (2022). Immunity to SARS-CoV-2: What Do We Know and Should We Be Testing for It?. J. Clin. Microbiol..

[9] Chen M., Zhang X.E. (2021). Construction and applications of SARS-CoV-2 pseudoviruses: A mini review. Int. J. Biol. Sci..

[10] Cai Z., Kalkeri R., Zhu M., Cloney-Clark S., Haner B., Wang M., Osman B., Dent D., Feng S.-L., Longacre Z. (2024). A pseudovirus-based neutralization assay for SARS-COV-2 variants: A rapid, cost-effective, BSL-2–based high-throughput assay useful for vaccine immunogenicity evaluation. Microrganisms.

[11] Keech C., Albert G., Cho I., Robertson A., Reed P., Neal S., Plested J.S., Zhu M., Cloney-Clark S., Zhou H. (2020). Phase 1-2 Trial of a SARS-CoV-2 Recombinant Spike Protein Nanoparticle Vaccine. N. Engl. J. Med..

[12] Sholukh A.M., Fiore-Gartland A., Ford E.S., Miner M.D., Hou Y.J., Tse L.V., Kaiser H., Zhu H., Lu J., Madarampalli B. (2021). Evaluation of Cell-Based and Surrogate SARS-CoV-2 Neutralization Assays. J. Clin. Microbiol..

[13] Bhiman J.N., Richardson S.I., Lambson B.E., Kgagudi P., Mzindle N., Kaldine H., Crowther C., Gray G., Bekker L.G., Novavax Trial Clinical Lead Author Group (2023). Novavax NVX-COV2373 triggers neutralization of Omicron sub-lineages. Sci. Rep..

[14] Fong Y., Huang Y., Benkeser D., Carpp L.N., Anez G., Woo W., McGarry A., Dunkle L.M., Cho I., Houchens C.R. (2023). Immune correlates analysis of the PREVENT-19 COVID-19 vaccine efficacy clinical trial. Nat. Commun..

[15] Wang L., Mohlenberg M., Wang P., Zhou H. (2023). Immune evasion of neutralizing antibodies by SARS-CoV-2 Omicron. Cytokine Growth Factor. Rev..

[16] Chen C., Liang J., Hu H., Li X., Wang L., Wang Z. (2023). Research progress in methods for detecting neutralizing antibodies against SARS-CoV-2. Anal. Biochem..

[17] Riepler L., Rossler A., Falch A., Volland A., Borena W., von Laer D., Kimpel J. (2020). Comparison of Four SARS-CoV-2 Neutralization Assays. Vaccines.

[18] US Food and Drug Administration Updated COVID-19 Vaccines for Use in the United States for 2023–2024. https://www.fda.gov/emergency-preparedness-and-response/coronavirus-disease-2019-covid-19/covid-19-vaccines-2023-2024#:~:text=The%20FDA%20has%20approved%20and,19%20caused%20by%20circulating%20variants.

[19] World Health Organization Statement on the Antigen Composition of COVID-19 Vaccines. https://www.who.int/news/item/18-05-2023-statement-on-the-antigen-composition-of-covid-19-vaccines.

