# Supplementary material for: Validation of a Pseudovirus Neutralization Assay for Severe Acute Respiratory Syndrome Coronavirus 2: A High-Throughput Method for the Evaluation of Vaccine Immunogenicity
Source: Microorganisms. 2024 Jun 14;12(6):1201. doi: 10.3390/microorganisms12061201 (PMC11205394; doi:10.3390/microorganisms12061201)
Supplement: Supplementary file 1 [file microorganisms-12-01201-s001.zip › microorganisms-3026277-supplementary.pdf]

microorganisms MDPI

SUPPLEMENT:

Supplementary Table S1. Pseudovirus assay results for SARS-CoV-2 Omicron variant strains.

**Parameter**	**Omicron BA.5**	**Omicron XBB.1.5**
Precision (total, intra-assay, and inter-assay)	≤50% GCV in 95%, 100%, and 97.5%	≤50% GCV in 80%, 97.5%, and 95%
Linearity	R^2 = 0.987 and 0.984	R^2 = 0.981 and 0.989
LLoQ	36	37
ULoQ	≥ 15,856	≥ 7561
Specificity	Pre-COVID samples: 100% <LLoQ<br>RSV-vaccinated: 100% <LLoQ<br>Influenza vaccinated: 100% <LLoQ	Pre-COVID samples: 100% <LLoQ<br>RSV-vaccinated: 100% <LLoQ<br>Influenza vaccinated: 100% <LLoQ

GCV—geometric coefficient of variation; LLoQ—lower limit of quantitation; RSV—respiratory syncytial virus; ULoQ—upper limit of quantitation.
